# Content-based user classifier to uncover information exchange in disaster-motivated networks

**DOI:** 10.1371/journal.pone.0259342

**Published:** 2021-11-16

**Authors:** Pouria Babvey, Gabriela Gongora-Svartzman, Carlo Lipizzi, Jose E. Ramirez-Marquez

**Affiliations:** 1 School of Systems and Enterprises, Stevens Institute of Technology, Hoboken, NJ, United States of America; 2 Information Systems, Carnegie Mellon University, Pittsburgh, PA, United States of America; University of South Carolina, UNITED STATES

## Abstract

Disasters strike communities around the world, with a reduced time-frame for warning and action leaving behind high rates of damage, mortality, and years in rebuilding efforts. For the past decade, social media has indicated a positive role in communicating before, during, and after disasters. One important question that remained un-investigated is that whether social media efficiently connect affected individuals to disaster relief agencies, and if not, how AI models can use historical data from previous disasters to facilitate information exchange between the two groups. In this study, the BERT model is first fine-tuned using historical data and then it is used to classify the tweets associated with hurricanes Dorian and Harvey based on the type of information provided; and alongside, the network between users is constructed based on the retweets and replies on Twitter. Afterwards, some network metrics are used to measure the diffusion rate of each type of disaster-motivated information. The results show that the messages by disaster eyewitnesses get the least spread while the posts by governments and media have the highest diffusion rates through the network. Additionally, the “cautions and advice” messages get the most spread among other information types while “infrastructure and utilities” and “affected individuals” messages get the least diffusion even compared with “sympathy and support”. The analysis suggests that facilitating the propagation of information provided by affected individuals, using AI models, will be a valuable strategy to pursue in order to accelerate communication between affected individuals and survival groups during the disaster and aftermath.

## Introduction

Disasters affect communities all around the world, as they can appear suddenly, without any warning, and cause immense damage. In this respect, communities’ response and recovery to disasters are related to their preparedness [1] throughout the stages of a disaster (i.e. the resilience of the community). During these events, affected people, concerned authorities, and volunteers search for actionable information to aid in damages and rescue the injured. Volunteer and governmental agencies need timely and credible information to save lives and get access to the people affected [2]. As such, social media has become an important tool to partially understand the social perspectives and needs before, during, and after a disaster [3, 4], and to extract first-hand information about the phases of the event.

Social media also contains information (in the form of messages, posts, blogs, etc.) about infrastructure and utility damages, donation efforts, regulatory guidelines, among many others. Therefore, it has become relevant to analyze social media for disaster management and to develop techniques that will aid decision-makers and first responders during and after a natural disaster strikes.

Work has been done to identify disasters and specific event messages within their time-frame to deploy aid [5–7]. In [8], Imran et al. surveyed computational methods to process social media messages and highlight both their contributions and shortcomings. These works have come across multiple challenges, among them, identifying which sources are credible and how to extract useful information for disaster risk management [9–12]. Common techniques for finding useful information involve topic modeling and sentiment analysis in social media [13–15]. During the last few years, deep learning models have also gained more reliable and accurate results [16, 17].

One important question that has not been carefully investigated is: “Do social networks efficiently connect affected individuals to disaster relief agencies?”. The analysis results on the tweets associated with hurricane Sandy evidence that tweets can enhance situational awareness during a humanitarian crisis and the speed at which information disseminates in social media during a disaster can help or hinder the impact of emergency response and aid [18]. Similarly, the results in [8] reported a higher level of user participation for tweets with information related to situational awareness, therefore demonstrating that social media is an important platform for coordinating future disaster mitigation.

The current literature shows the importance of disseminating information during a disaster [1, 2, 7, 8, 19, 20], but there is still research to be done in terms of the speed, accuracy and diffusion rates that can be measured during a disaster. This work addresses the following research questions:

- Which electronic media based information is useful and accurate during a disaster and how do we identify it?
- Most algorithms to answer the first question require labels (supervised learning) which are
  1. hard to come by in real-time or
  2. create after a disaster has occurred.
- Most algorithms to answer (2) require labels (supervised learning), that are hard to come by in real-time or even create after a disaster has occurred. For us to eventually have real-time (or at least faster actionable, faster) information during a disaster we need algorithms that can be pre-trained and do not rely on labels.
- For us to eventually have real-time (or at least faster actionable) information during a disaster we need algorithms that can be pre-trained and do not rely on labels.
- How do we apply NLP-related algorithms to disaster mitigation?
- How can we measure this information without relying on labels?
- How can we increase the accuracy of the information we obtain during disasters?
- How can we measure the diffusion of different types of information during a disaster?

The analysis on Twitter data from hurricanes Dorian and Harvey performed in this paper confirms the observations reported in [18] and shows the tweets about the affected individuals and damaged infrastructures gets the least diffusion rate among the other groups including sympathy and supports. This calls for the need of developing AI-models to facilitate the propagation of the information online during the disaster. In order that, cross-disaster AI models need to be developed and trained in advance to be used online during the disaster.

This work deploys the BERT model [21] to classify the disaster-motivated posts on social media. The model is first fine-tuned using a historical cross-disaster dataset in the literature [22] and then is evaluated using the collected tweets from hurricanes Dorian and Harvey (in this study tweets have been used as defacto social media messages). And alongside, the network between the members of the “disaster-motivated community” is constructed based on the retweets and replies on Twitter. In this manuscript, the term disaster-motivated community is used to represent the set of all the users who sent at least one tweet or reply including one of the disaster-specific hashtags during the disaster. Finally, PageRank score [23] of the nodes in the constructed network are used to measure the diffusion rate of each type of disaster-motivated information in the network. The results show that the “cautions and advice” messages get the most spread among other information types while “infrastructure and utilities” and “affected individuals” messages get the least diffusion.

The proposed framework has the following contributions:

(1) By identifying more accuracy in classifying disaster-motivated tweets using the BERT algorithm, a methodology is introduced to use state-of-the-art NLP models to characterize disaster-motivated information diffusion on social media.
(2) The interaction (replies and retweets) between the social media users who share and follow the disaster-motivated information is used to create a disaster-motivated network of users. The process is explained using the dataset from hurricanes Harvey and Dorian.
(3) The PageRank algorithm is used to measure the diffusion rate of different types of information in a disaster-motivated network. The diffusion rate of different information types are measured and compared for hurricanes Harvey and Dorian.

The remainder of the paper is organized as follows: section “Related Works” describes the previous works done on disaster-specific text classification methods as well as the major studies on characterizing the diffusion rate of different types of information on social media. Section “Methodology” describes the proposed framework; explains how the fine-tuned BERT model is used to categorize disaster-specific tweets and how the diffusion rates of different categories are measured on social media. The section “Case Study” reports the analysis results on the tweets associated with hurricanes Harvey and Dorian. Finally in “Conclusions and Discussions” the results, possible applications of this study, and the future works are discussed.

## Related works

### Disaster-specific text classification methods

One of the important contents of natural disaster emergency decision lies in the way to describe the data with different sources, data mapping and fusion, feature extraction and classification, quick and accurate access to valuable information and intelligent decision in an emergency response [24]. In combination with other communication methods, the combined use of social media and social media analysis tools provides humanitarian actors with an opportunity to increase the effectiveness of their social media communication on disaster preparedness [25]. Other approaches include using mathematical models and heuristics to identify information dissemination in social media networks [26], or using the same heuristics to determine node optimization in social networks and gather insights on user’s behavior [27].

A major concern over the last decade has been identifying crisis events and disasters in social media [28–30]. This identification, and further classification for disaster management, requires proper training of machine learning algorithms.

For this purpose, different authors have deemed it necessary to first collect and build appropriate repositories for exploration. McMinn, Moshfeghi and Jose [6] collected 120 million tweets for training and exploration purposes. Only 150,000 of these tweets are labeled and not all tweets correspond to crisis events, but just events happening in social media. Later on, Olteanu, et al. [22] collected crisis specific tweets, as they create a lexicon revolving around crisis situations for training and automatic detection of crisis topics on Twitter. Their methodology shows great potential and improvement above manually labeled tweets. A couple of years later, Imran, Mitra and Castillo [11] took the previous concepts to create a repository of 19 different crises events represented as Twitter corpora from 2013 to 2015, that have been human-annotated to train word2vec word embeddings. Recently, Huang et al. [31] introduced an automated tweet extraction approach by mining both visual and textual contents in the tweets. The method then was applied to the Houston flood as a case study to label on-topic tweets [32].

Once repositories and lexicons have been created, the next challenge is identifying informative information when disasters occur; i.e., information useful for decision-makers and first-responders when a disaster strikes. Traditional machine learning techniques were first explored, but they do not deliver the necessary accuracy in several situations, from using decision trees [10, 14] to unsupervised topic modeling techniques [9].

During the past five years, Convolutional Neural Networks (CNNs) have been explored for the accuracy of information during disasters detected through Twitter. Caragea, Silvescu and Tapia [33] suggested using CNNs to extract accurate information from Twitter to aid first responders during flooding events. CNNs proved to have performed significantly better than when utilizing traditional Bag of Words, Naive Bayes and Support Vector Machines. Burel and Alani [34] created Crisis Event Extraction Service (CREES) to automatically classify tweets during crisis or disaster events. CREES is based on CNNs, to classify text in three ways: crisis versus non-crisis, type of crisis, and type of information. Recently, other authors suggest taking CNNs further and exploring Deep Convolutional Networks to identify informative tweets and to place them into classes [16]. Even though CNNs showed a good performance with F1 scores higher than 75% in some cases, while the training data is small (a few hundred) they under-perform compared to traditional machine learning methods such as Naive Bayes, CART and Support Vector Machines (SVMs) [35]. Moreover, CNN models showed lower performance on classifying tweets compared to news articles [36] with a more uniform style and tone.

Deep learning models usually require to be trained from the ground up with a large amount of labeled data, thereby they are not suitable for real-time predictions. Recently transfer learning has led to an improvement of the performance of the deep learning models with a small amount of data [37]. Models like BERT, are first pre-trained via a large dataset to learn a wide range of general information about a language. Then, a small dataset (several hundreds of labeled messages) is needed to fine-tune the model for different Natural Language Processing (NLP) tasks [21].

Experiment results [37–39] on comparing BERT with traditional machine learning models suggest that using BERT (or other models that benefit from transfer learning) the performance of NLP models on disaster datasets can be improved significantly. Recent work on applying BERT models to social media for COVID-related topics has yielded insightful results in terms of information dissemination and community identification [40–43]. This leads to the belief that BERT models can also be successful with identifying relevant information for disasters such as hurricanes. Miyazaki, et al. [44] began exploring BERT models for classification in annotated disaster tweets.

Jain et al. [45] compared the performance of BERT with previously developed NLP models on a group of disaster-specific classification benchmarks and their analysis showed BERT had no advantage over the other previous NLP methods like ELMO, Word2Vec, and GloVe. Maharani [46] used the BERT for sentiment analysis of the tweets associated with Jakarta flood for emergency responses and situational awareness. Fan et al. [47] used the BERT model to filter out off-topic posts that may impair the precision of unfolding disaster events, the remaining tweets then geo-tagged using Named Entity Recognition and Google Map Geocoding API. BERT [21] is a new methodology and yet to be extensively applied to disaster management through social media, and is therefore explored in this research.

Though several studies have been conducted on using NLP models to classify disaster-motivated tweets, few works have been done on further analysis using NLP results to measure the diffusion of different types of information on the social media during the disaster and its aftermath.

### Diffusion rate of different types of disaster-specific information on social media

Different approaches have been introduced to characterize the information diffusion patterns on social networks, using contagion probabilistic models [48, 49] or conditional coordination games [50]. And several algorithms are developed to maximize or minimize the information diffusion in the network [51], including: (1) maximizing the information flow for influence maximization based on location [52–54] and topic [55, 56], and (2) minimizing the information diffusion for misinformation influence minimization [57]. However, there are few studies on characterizing the information diffusion during and in the aftermath of disasters. Analysis of Twitter data associated with Hurricane Sandy in [18] showed that the speed of diffusion is contingent upon the type of users that originally publish this information. When information is shared by users with higher influence, a higher number of followers, it will diffuse faster than while the originators’ influence is limited. This is especially the case for disasters, while the majority of the affected individuals by disasters are located at the periphery of the social network with minimal impact in the spreading process through the core of the network [58].

As a side note, engineering the information flow is prone to different types of biases due to epistemic uncertainties, and this may raise fairness challenges. In [59], Bolzern et al. showed how algorithmic filtering of contents exchanged on social networks, even an unbiased neutral intervention, can favor one faction just by tuning the interaction intensity.

## Methodology

**Fig 1:**
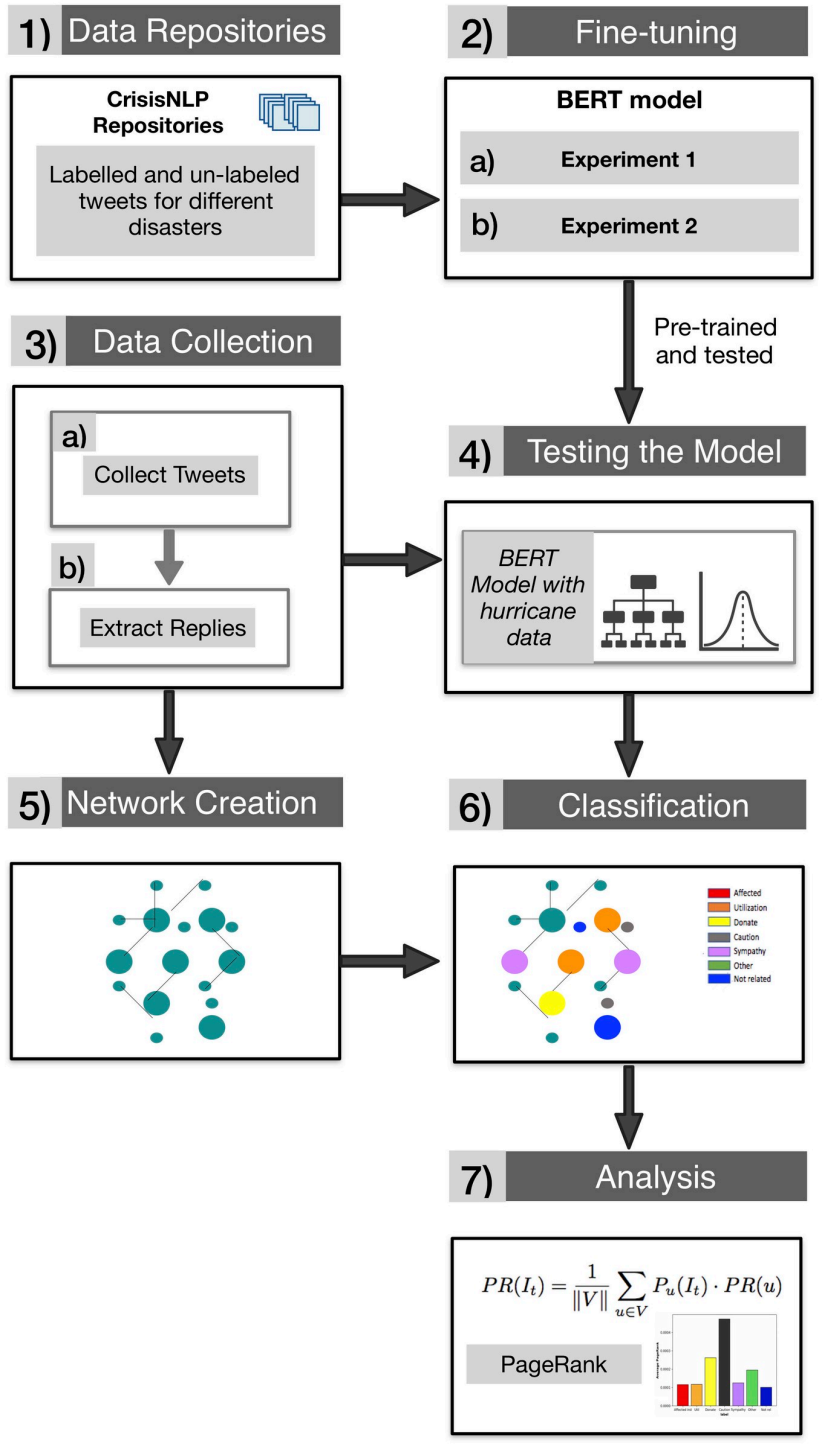
Methodology overview. The process includes fine-tuning the BERT model using the datasets in the literature, collecting new datasets to validate the fine-tuned model, as well as creating the network between users in the disaster-motivated network and evaluating the spread of each type of information in the network.

**Fig 2:**
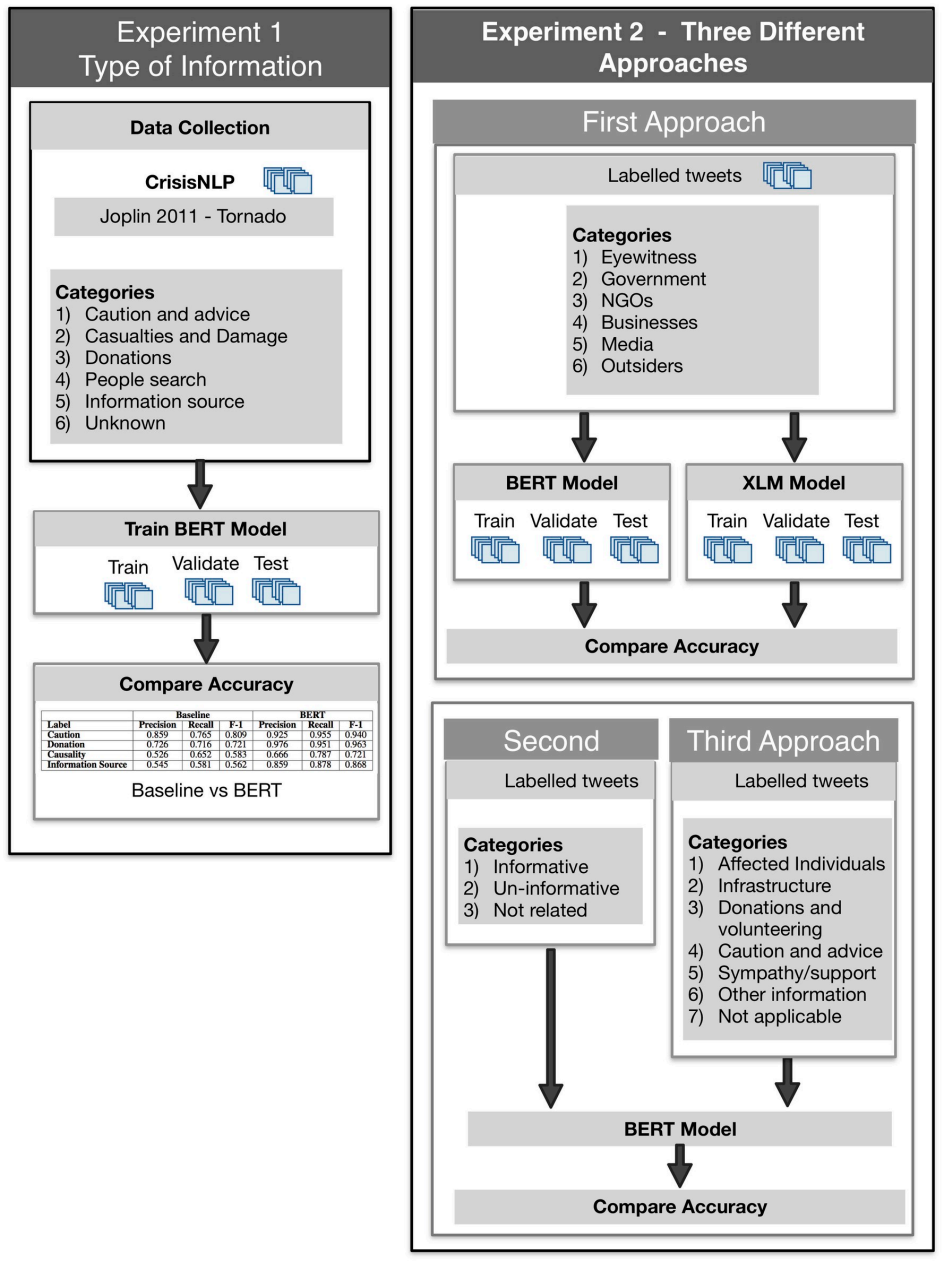
Overview description of fine-tuning process. According to the results from the first experiment the fine-tuned model shows a significant improvement compared to the previous methods. In the second experiment, confusion matrix is used to evaluate the model performance.

The overall proposed methodology (the framework) followed in the study can be viewed in Fig 1 and consists of the following steps:

1. The first step involves gathering tweets from the CrisisNLP [60] repositories, used by previous authors [7, 11, 61]. These tweets belong to previous disasters (hurricanes, floods, tornadoes, etc.), and a subset of them have been labeled. These will serve as a baseline for fine-tuning and testing the model developed in this study.
2. The second step involves using the tweets from step 1 to fine-tune the classifier model, through two different experiments, detailed by Fig 2.
  (a) corresponds to Experiment 1.
  (b) corresponds to Experiment 2. As seen in Fig 2, experiment 2 implements three different approaches to classifying the tweets based on (i) type of actors, (ii) the type of information obtained from the tweets, (iii) and how informative the tweets are.
3. The third refers to the collection of tweets for hurricane Dorian (2019) and hurricane Harvery (2017), based on event-specific hashtags and keywords. This step involves two sub-steps:
  (a) An initial seed of tweets are collected for the disaster. The tweets posted from two weeks before the hurricane stroke until two weeks after the hurricane ended and contain one of the proper hashtags of each hurricane were collected.
  (b) From the initial seed (previous sub-step) of tweets their replies and retweets were extracted.
4. Step 4 involves using the pre-trained BERT model from step 2 on the tweets collected on step 3.
5. Step 5 uses the tweets collected in step 3 to create a network. The retweets and replies are used to generate a network between actors, where the node *V* is connected to the node *U* by a link, if at least one of the tweets posted by *U* is retweeted or replied by *V*.
6. Step 6 uses the classification output from step 4 to classify the nodes in the network created in step 5. Each actor is classified according to the type of information provided by her tweets and color is used to visualize the interaction between different actors in the disaster-motivated network.
7. Finally, the network classified in step 6 is analyzed to determine the diffusion rates of different types of information and different types of actors. To measure the diffusion rates PageRank algorithm is used as a baseline.

In the remainder of this section, the steps of the process are explained in more detail.

### Data repositories and data collection

There are two main data repositories used in this research. The first one contains Twitter data previously collected by CrisisNLP [60] repositories, and previously used by authors [7, 11, 61]. These tweets belong to previous disasters (hurricanes, floods, tornadoes, etc.), and a subset of them have been labeled. These will serve as a baseline for fine-tuning and testing the model developed in this study.

The second data repository was collected to provide a case study for this research. Tweets were extracted through a public Twitter API using *twarc*—a Python-based command-line tool to archive Twitter data. The collection method complied with the terms and conditions for Twitter Developer Agreement. For hurricanes Dorian and Harvey, the proper event-specific hashtags (#dorian, #hurricanedorian, #bahamas for hurricane Dorian, #hurricaneharvey and #Harvey for hurricane Harvey) were used to collect the disaster-specific tweets. All the tweets that contain at least one of the above hashtags and were posted two weeks before each hurricane landing, up until two weeks after the hurricanes had ceased. Thus, the collected tweets for hurricanes Dorian in the dataset were posted from August 20th, 2019 until September 24th, 2019. While, for hurricane Harvey the collected tweets belong to the time interval of August 14th, 2017 until September 14th, 2017. The collected dataset is available as tweet IDs on http://github.com/pbabvey/DisasterTweets.

In the next two subsections, the process of fine-tuning the BERT model for tweet classification based on the type of information and the type of actors are explained.

#### Experiment I: Information types

A subset of the data source Crisis NLP website [60] is used to fine-tune the BERT model for tweet classification based on the information type. The tweet corpus refers to the Joplin 2011 tornado that struck Joplin, Missouri in May 2011 and released by [61]. The study used an ontology developed by Vieweg et. al [7], to classify the disaster-motivated messages. The ontology is based on a combination of data-driven analysis of Twitter communications combined with findings from the disaster literature and official government procedures for disaster response. Based on this ontology the following categories were defined [11, 61]:

- Caution and advice: if a message conveys/reports information about some warning or a piece of advice about a possible hazard of an incident.
- Casualties and damage: if a message reports the information about utility or infrastructure damage done by an incident.
- Donations of money, goods or services: if a message speaks about money raised, donation offers, goods/services offered or asked by the victims of an incident.
- People missing, found, or seen: if a message reports about the missing or found person effected by an incident or seen a celebrity visit on ground zero.
- Information source: if a message conveys/contains some information sources like photo, footage, video, or mentions other sources like TV, radio related to an incident.

A total of 3,438 labeled tweets used to fine-tuned the BERT classifier. The BERT model is then compared against the precision, recall and F-1 scores already obtained by the authors [61] on the same dataset. The results of the proposed BERT-based model and the baseline are shown in Table 1.

**Table 1:** Performance comparison of BERT against baseline results. As can be seen the performance is significantly improved.

	Baseline			BERT		
Label	Precision	Recall	F-1	Precision	Recall	F-1
**Caution**	0.859	0.765	0.809	0.925	0.955	0.940
**Donation**	0.726	0.716	0.721	0.976	0.951	0.963
**People**	0.526	0.652	0.583	0.666	0.787	0.721
**Information Source**	0.545	0.581	0.562	0.859	0.878	0.868

#### Experiment II: Informativeness of posts, information types, and actor types

In this part of the methodology, the BERT model is fine-tuned to classify the tweets based on three different criteria of informativeness, information type, and actor type. A study by Olteanu et al. [22] is taken into consideration. A total of 24,177 tweets were labeled in this dataset associated with their study based on the three different criteria. Among them, some tweets were in languages other than English, such as Spanish and Russian. The extracted information is a cross-disaster dataset containing a group of 26 disasters. The dataset is divided into training, validation, and test with 70%-10%-20% ratios.

According to the informativeness criteria, the posts are divided into three categories of: informative, un-informative, and not related. The accuracy of the BERT-based model was 88% and the confusion matrix can be found in S1 Fig.

According to the information type criteria, the tweets are divided into six different categories. The information types, as well as the most frequently used words for each category are represented in Table 2. The stop words—most common, short function words, such as the, is, at, which—were removed from the lists. The accuracy of the model to classify based on the information type was 78% (compared to 16% accuracy of random assignment of the tweets to six categories).

**Table 2:** Most frequent words of different information types.

Information type	Most frequent words and bi-grams
**Affected individuals**	injured people, dead, killed, fire, death toll, rescue
**Infrastructure and utilities**	building collapse, train derailment, plant explosion, helicopter crash, closed, water, fire, flood, homes, Refinería, oil, roof damage
**Donations and volunteering**	raise funds, emergency, evacuation centers, help, relief efforts, support, food, water, victims, accepting donations, aid, rescue, shelter
**Caution and advice**	breaking news, typhoon, tropical storm, weather bulletin, rains, metro, hits, alert, update, warning, stay at, emergency, flooding, tsunami, earthquake
**Sympathy and support**	please pray, keep safe, god bless, affected, hope, people, families, tragedy, sad
**Other useful information**	photo of, press conference, report, meteor, train

According to the actor type criteria, the tweets are divided into seven different categories. The outlined types of actors are as follows:

- Eyewitness: citizen reporters, local individual
- Government: authorities, police, public service agencies
- NGOs: non-profit or non-governmental organizations
- Businesses: commercial organizations, enterprises, for-profit corporation
- Media: news organizations
- Outsiders: sympathizers, distant witness, non-locals

**Fig 3:**
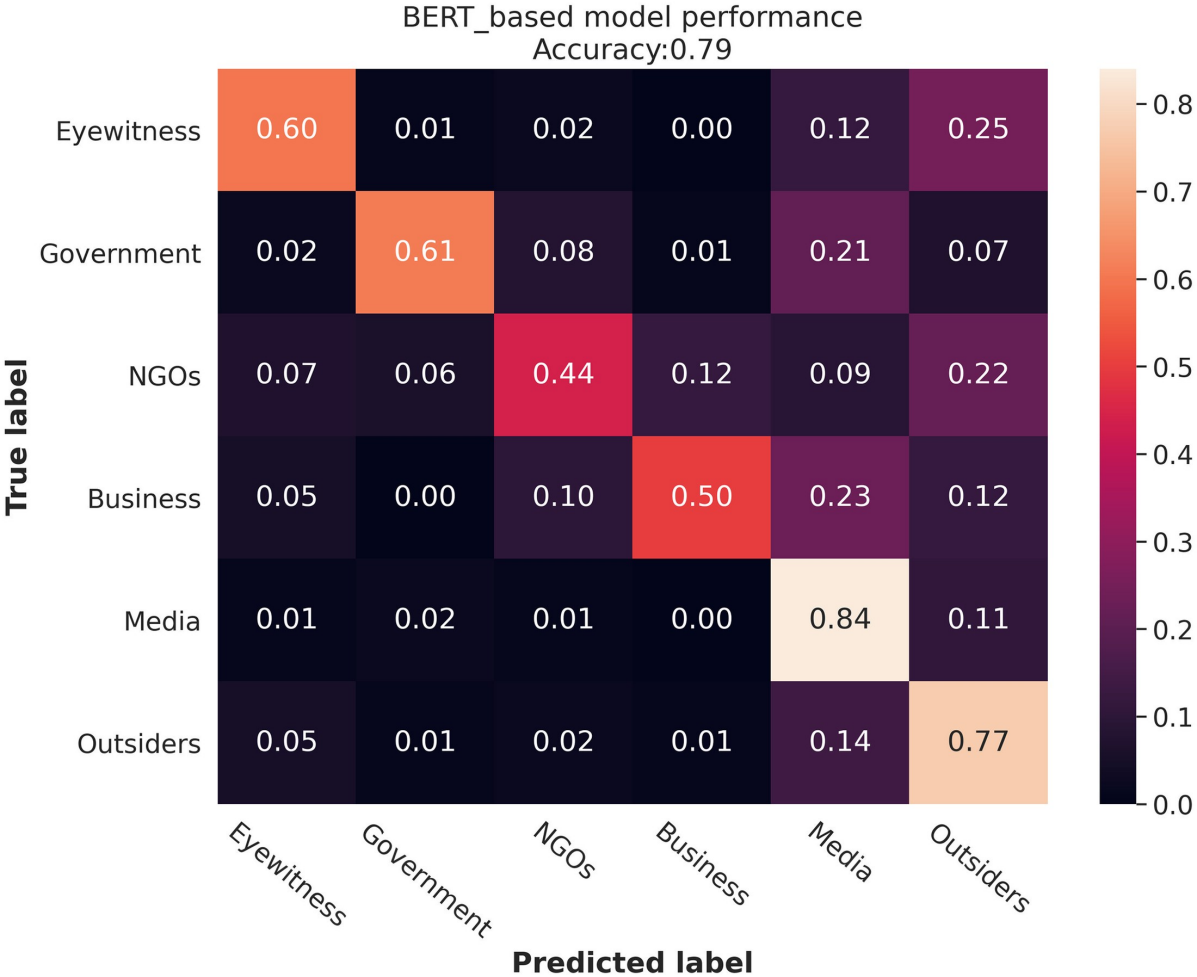
Confusion matrix of BERT model for the type of actor classification. The model shows a better performance in detecting the posts from Media, Outsiders, Eyewitnesses, and Government, while the performance is lower for messages from NGOs and Business.

The accuracy of the BERT-based model was 79%. Fig 3, displays a confusion matrix showing the performance of the BERT model.

### Network creation and diffusion rates

The replies and retweets on Twitter were used to generate a network between users in a disaster-motivated community. In this network, each node represents a user who posted at least one disaster-motivated tweet, and a user *v* receives links from a user *u* who retweeted or replied at least one of the posts sent by *v*.

It is worth noting that the diffusion rate of information through the disaster-motivated network may not be proportional to the diffusion rate of that information in the whole Twitter network in general. For example, tweets posted by a politician or a public figure may get thousands of retweets and replies on Twitter, while it does not receive proportional replies or retweets in the disaster-motivated network. In this study, the diffusion rate of the messages in the disaster-motivated network is considered.

PageRank is used as a metric to measure the diffusion rate of the tweets posted by each user in the network. The PageRank score of a user represents the probability of ending up reading a tweet by a user in case someone started reading the disaster-motivated tweets. PageRank works by measuring the quality of links to a node to determine a rough estimation of how important a node is in the network [23]. PageRank assigns a score to each node in the network using a mathematical algorithm that satisfies the following equation for each node *v*:

$$\begin{array}{c} PR\left(v\right)=\frac{1-d}{\left\|V\right\|}+d\cdot \sum_{u\in N(v)}^{}\frac{PR(u)}{\left\|N(u)\right\|} \end{array}$$ (1)

In the above equation, *V* is the set of all the nodes in the network; *PR*(*v*) represents the PageRank score of the node *v* in the network; *d* is the residual probability and is usually set to 0.85. *N*(*v*) shows the set of all the nodes like *u*, that the node *v* receives a link from them. In synthesis, the PageRank score of a user *v* is equal to a constant plus the average of the PageRank scores of the users who retweeted or replied to the tweets of user *v*. The average PageRank score of the nodes is equal to one.

To extend the PageRank metric from users to a normalized PageRank score for information types, the following equation was used:

$$\begin{array}{c} PR\left(I_{t}\right)=\frac{1}{V}\sum_{u\in V}^{}P_{u}\left(I_{t}\right)\cdot PR\left(u\right) \end{array}$$ (2)

In the above equation, *I*_*t*_ is an information type, *V* is the set of all the nodes in the network, *PR*(*u*) represents the PageRank score of the node *u* in the network. *P*_*u*_(*I*_*t*_) shows the share of information type *I*_*t*_ from all the tweet posted by *u* in the dataset. A similar method is used to calculate the PageRank scores of different actor types.

This metric will be used in the network created for the disaster-motivated community for hurricanes Harvey and Dorian. First, the tweets will be classified in terms of type of information and type of actors. Then, the normalized PageRank scores will evaluate the diffusion rate of different information types and actor types during a disaster. The next section applies the full methodology to the second data source of tweets collected from hurricanes Harvey and Dorian.

## Case study: Hurricanes Harvey and Dorian

In this section, the fine-tuned classifier is used to classify the collected tweets from hurricanes Dorian and Harvey. For hurricanes Harvey and Dorian tweets from 131,000 and 144,000 users, respectively, were collected. These users sent at least one post using the disaster-specific hashtags (as discussed in step 3 and 4) and are regarded as disaster-motivated communities in this paper. To validate the performance of the fine-tuned classifier on the collected dataset from hurricanes Dorian and Harvey, a group of 1200 tweets was selected randomly and labeled with the same criteria for informativeness, type of information, and type of actor. The dataset was divided into training, validation, and test with 70%-10%-20% ratios. The fine-tuned model performed better with 75% accuracy for information type classification, 75% for actor type classification, and 87% for informativeness classification in comparison with the model without fine-tuning (on historical data) with 72%, 71%, and 83% respectively. Additionally, the accuracy of the fine-tuned model without retraining the new dataset (from hurricanes Dorian and Harvey) remained almost the same with 75% accuracy for information type classification, 74% for information source classification, and 86% for informativeness classification. These results show that transfer learning is an effective method to enable AI models to classify the online disaster-motivated tweets more accurately.

In the next step, the page rank score of different information types for hurricanes Dorian and Harvey are analyzed.

**Fig 4:**
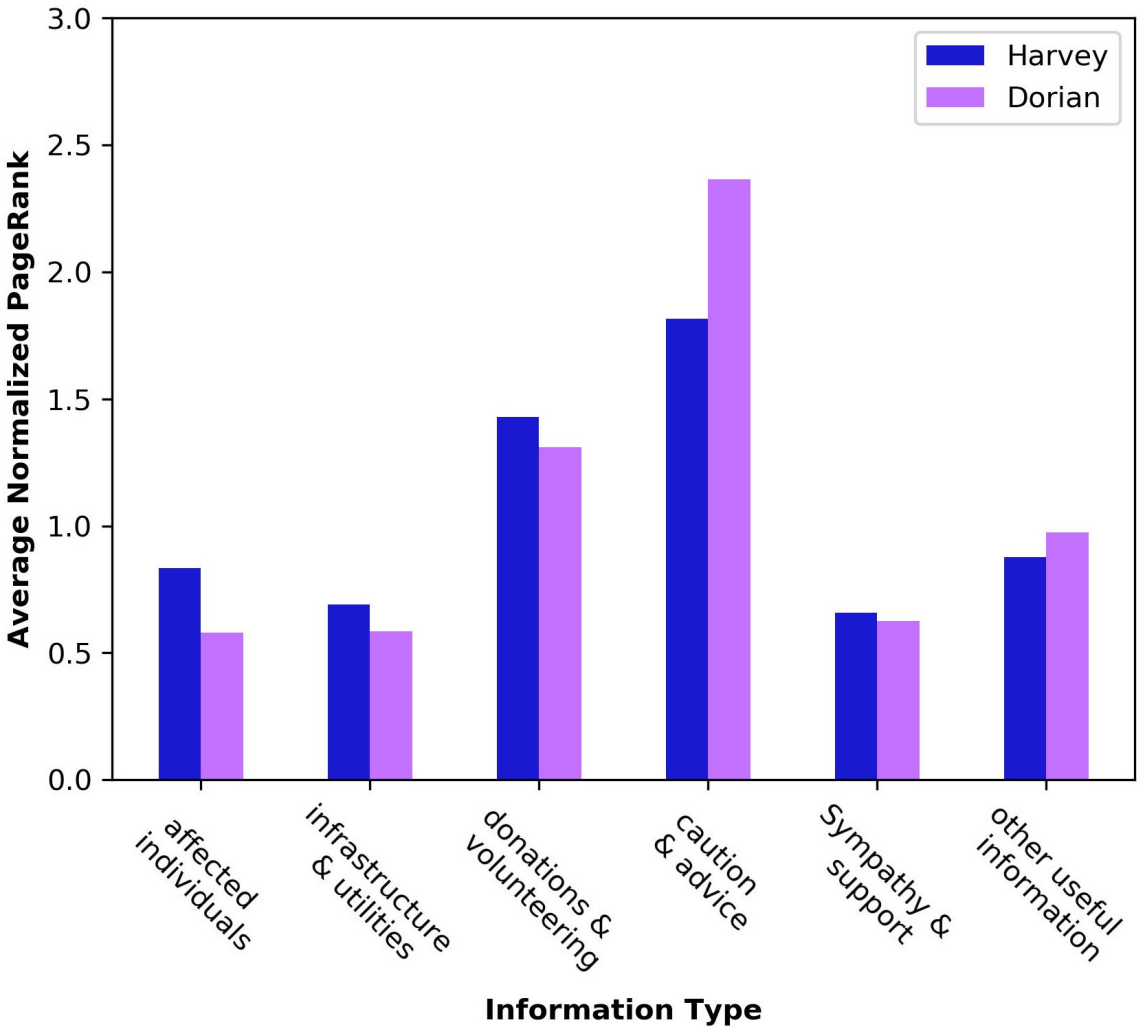
Average PageRank score of different information types. “Caution and advice” gets the highest diffusion rate in the network, followed by “Donations and volunteering” while the messages by “Affected individuals” do not get enough spread. In hurricane Harvey however, the diffusion rate of the messages by “Affected individual” and “Infrastructure and utilities” get more spread compared to hurricane Dorian.

Fig 4 shows the average PageRank scores for different information types for hurricanes Dorian and Harvey.

According to the results in Fig 4 the information about “Affected individuals” and “Infrastructures” gets the least diffusion rate among the disaster-motivated community in hurricane Dorian. The distribution of the scores is similar in both hurricanes, where the top scores categories are “Caution and advice”, and “Donation and volunteering”.

**Fig 5:**
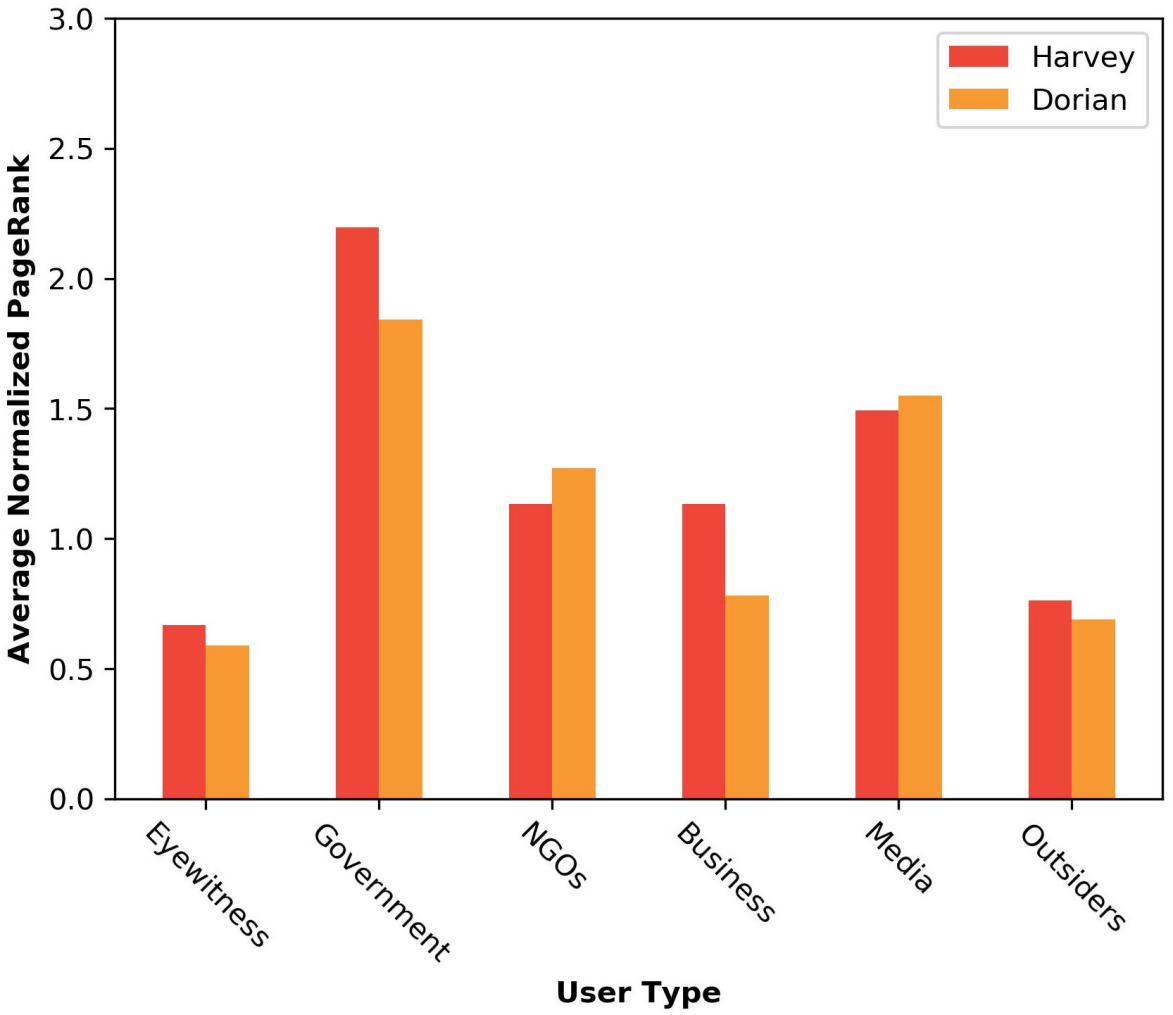
Average PageRank score of different user types. The messages by “Government” get the highest spread in the network, followed by messages “Media” while the messages by “Eyewitnesses” get the lowest spread among others. In hurricane Harvey however, the spread of the “Eyewitnesses” messages get more spread compared to hurricane Dorian.

Fig 5 displays the average PageRank scores for different actor types in the disaster-motivated community network.

Not surprisingly the messages by “Government” and “Media” get the most diffusion rate. In both cases, the tweets by “Eyewitnesses” get the least spread even in comparison with “Outsiders” who mainly post sympathy, critics, and political conversations. Hurricane Harvey displays more diffusion rate for the tweets posted by “Eyewitnesses” and “Businesses” in comparison with hurricane Dorian, where the most proportion of the attention is given to the “Caution and advice”.

To visualize the network between actors during the time of the disaster, all the users with lower than three disaster-motivated posts were filtered out. The size of the nodes is proportional to the PageRank score of them. The color of each user was determined based on the majority of actor type labels for their generated content (Hard voting was used to determine the type of a user, for example if a user posted eight disaster-specific tweets in total and among them, six tweets were labeled as eyewitness then the user is categorized as eyewitness).

**Fig 6:**
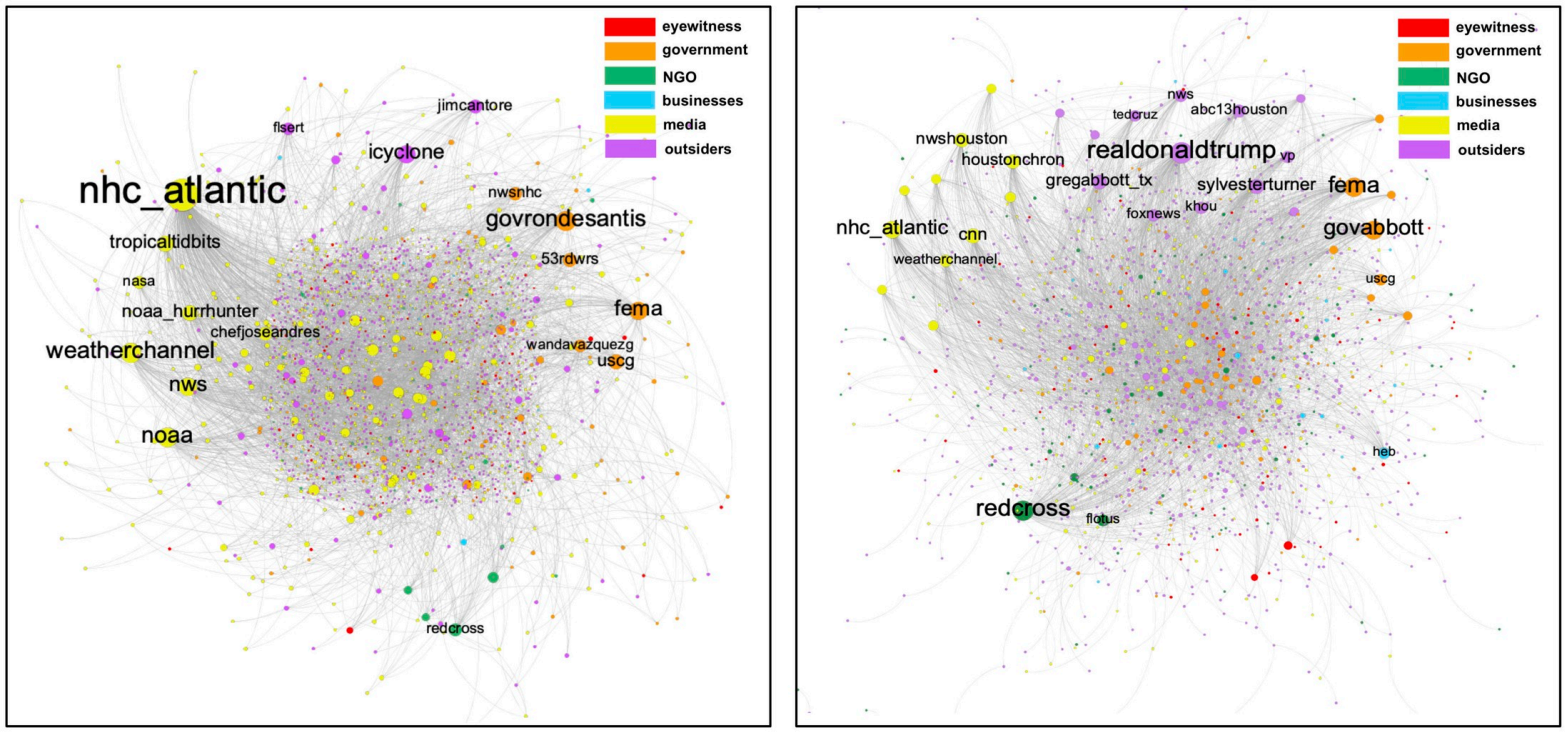
The network between users for A: hurricane Dorian B: hurricane Harvey. The size of the nodes are proportional with the PageRank score of them.

Fig 6a) and 6b) show the network between users in hurricanes Dorian and Harvey respectively.

In both networks the username of the most effective Twitter accounts are represented. As can be seen in the figure, the model is not completely successful in determining all the user types. The model classify users based on the provided content by each user and it may label some individuals, who actively seeking for fundraising, as NGO; or label a news agency who provides a multitude of retweets of politicians as an outsider. However, the model successfully detected “Eyewitnesses”, “Outsiders”, and the other user type groups. As can be seen, some meteorologists and hurricane chasers exist among the most contributing individuals in the network. For the sake of privacy, except for the public figures, the username of the individuals are not represented in the figure.

The network analysis results show that the users with the most diffusion rate in the network are mainly media, governmental agencies, and public figures. For hurricane Harvey, “Businesses” and “NGOs”, like *@redcross* and *@heb*, played a more important role in the network while in the hurricane Dorian the posts by “Media” and “Outsiders” (including hurricane chasers) got the most diffusion rate in the network. More importantly, in both cases the messages by “Eyewitnesses” get the least diffusion rate in the network. AI models can be used online to boost the propagation of eyewitnesses messages in the affected area through the network and help them to connect to the volunteers and survival groups.

Although numerous factors can lead to a difference in the PageRank averages, such as the population of the affected areas, the severity of the hurricane, and preparedness, such models can be exploited to compare the spread of each type of information in different disasters. The results also show that, in case of disaster, important messages informing about affected individuals or damaged structures may be shadowed by repetitive cautions, political discussions, and other non-informative messages.

## Conclusions and discussions

Social media is widely used in disaster response management. Millions of tweets are published both during and in the aftermath of crises. Some of these tweets reflect observations, while many others contain offers and requests for help. One important question that remained unexplored is that whether the social media efficiently connect affected individuals to disaster relief agencies, and if not, how AI models can facilitate information exchange between the two groups.

In this study, a new method is introduced to measure the diffusion rate of different information types on social media. The network between users is constructed based on the retweets and replies on Twitter, and alongside, the fine-tuned BERT classifier is employed to categorize tweets based on the information types and actor types. The extracted network along with classification enables the measurement of diffusion rate of different information types.

The methodology applied to the tweets associated with hurricanes Harvey and Dorian. For hurricane Harvey, the tweets by “Businesses” and “NGOs” got higher diffusion rates compared to hurricane Dorian. More importantly, in both cases, the “cautions and advice” messages got the most spread among other information types while “infrastructure and utilities” and “affected individuals” messages got the least diffusion even compared to “sympathy and support”. Also, the tweets by “Eyewitness” users get the least diffusion rates in the network followed by “Outsiders”, and On the other hand, the tweets by “Governments” and “Media” get the highest diffusion rates in the network. Comparing the results for the two hurricanes, in hurricane Harvey the tweets by “Eyewitnesses” and “Businesses” get higher attention compared to hurricane Dorian.

These results affirms that important messages informing about affected individuals or damaged structures are shadowed by repetitive cautions, political discussions, and other non-informative messages. This calls for the need of developing AI-models to facilitate the propagation of the information provided by affected individuals during the disaster.

From a methodological point of view, the limitations have to be discussed as they can be translated, in part, into future works. The developed model in this study benefits from supervised learning and is trained based on a cross-disaster dataset. Although the model shows a reliable performance on the dataset from hurricanes Dorian and Harvey with the majority of English language tweets, before extending the application of the model to other cases, further analysis is needed to evaluate the transferability of the model to other disasters rather than hurricanes and in low resource languages.

Future work involves developing a model as an adaptive classifier in real-time (while the disaster is happening) to categories the tweets based on the information type and foster the information spread provided by affected individuals and to mobilize help to areas of need and evaluate how the algorithm works under these circumstances.

## Supporting information

S1 Fig Confusion matrix of BERT model on informativeness classification. The model shows a good performance in detecting the informative posts.
(TIF)

S2 Fig Confusion matrix of BERT model on information type classification. (TIF)
