# Supplementary figures and images for: Content-based user classifier to uncover information exchange in disaster-motivated networks
Source: PLoS One. 2021 Nov 16;16(11):e0259342. doi: 10.1371/journal.pone.0259342 (PMC8594803; doi:10.1371/journal.pone.0259342)

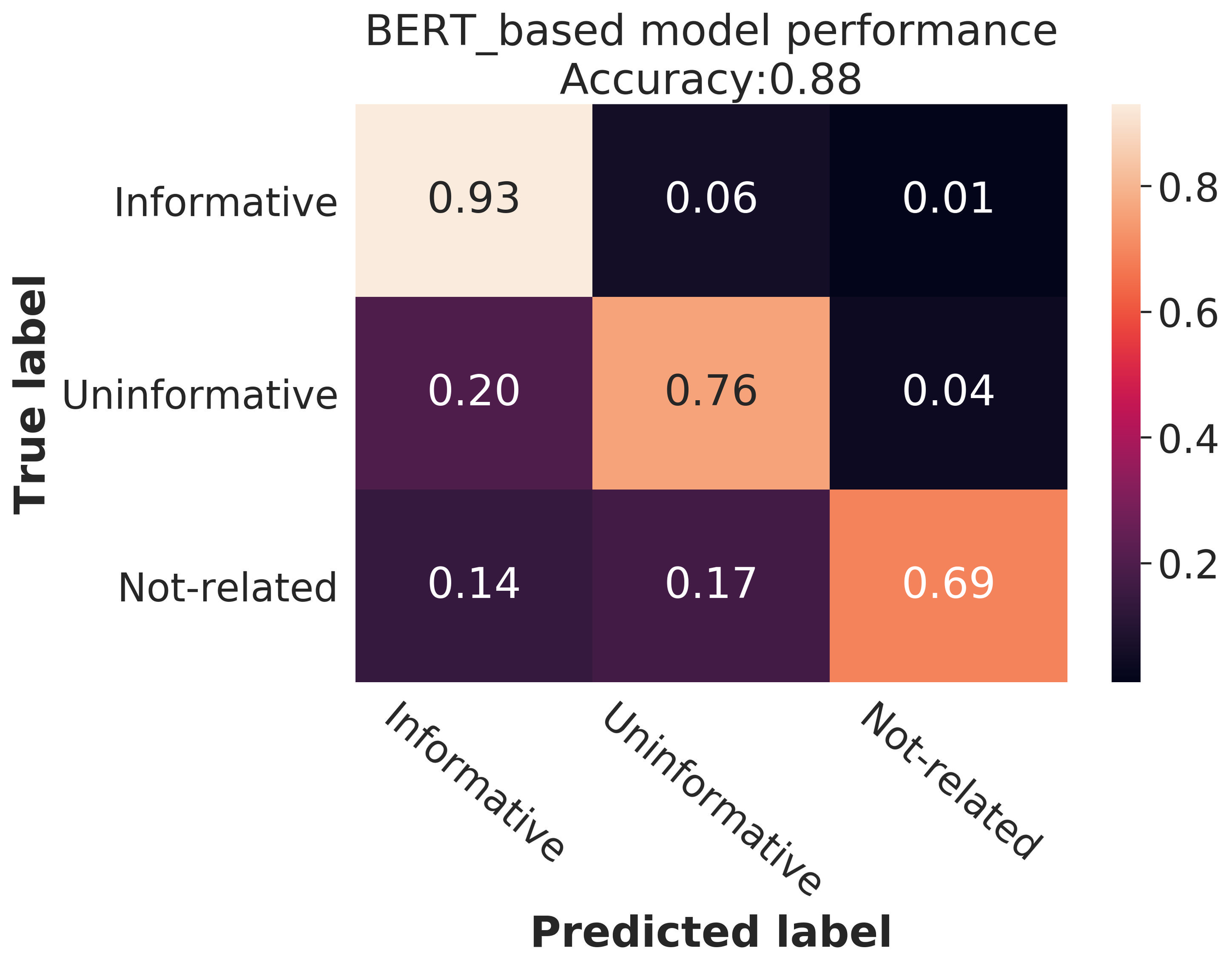

Supplement: S1 Fig — The model shows a good performance in detecting the informative posts. (TIF) [file pone.0259342.s001.tif]

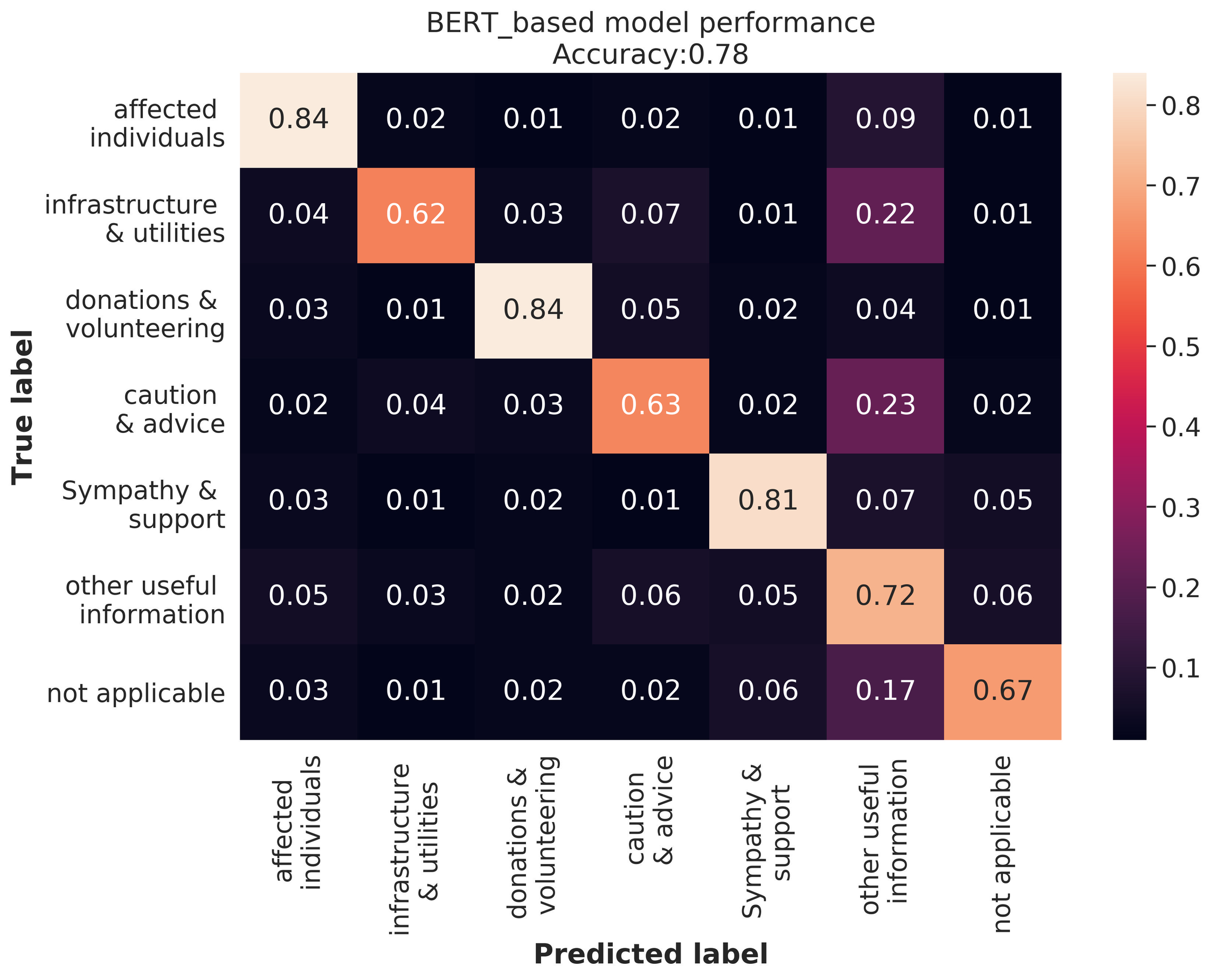

Supplement: S2 Fig — (TIF) [file pone.0259342.s002.tif]
